# Mammary Stem Cells in Domestic Animals: The Role of ROS

**DOI:** 10.3390/antiox8010006

**Published:** 2018-12-26

**Authors:** Mario Baratta, Silvia Miretti, Elisabetta Macchi, Paolo Accornero, Eugenio Martignani

**Affiliations:** Department of Veterinary Science, University of Turin, Largo Braccini 2, 10095 Grugliasco (TO), Italy; silvia.miretti@unito.it (S.M.); elisabetta.macchi@unito.it (E.M.); paolo.accornero@unito.it (P.A.); eugenio.martignani@unito.it (E.M.)

**Keywords:** ROS, stem cell, mammary gland, bovine, regenerative involution

## Abstract

Reactive oxygen species (ROS) are produced as a natural byproduct of the normal metabolism of oxygen and play significant roles in cell signaling and homeostasis. Although ROS have been involved in pathological processes as diverse as cancer, cardiovascular disease, and aging, they may to exert an effect even in a physiological context. In the central nervous system, stem cells and hematopoietic stem cells are early progenitors that contain lower levels of ROS than their more mature progeny. These different concentrations have been reported to be crucial for maintaining stem cell function. Mammary gland remodeling has been proposed to be organized through the activation and regulation of cells with stemness, either considered real stem cells or primitive precursors. Given the state of oxidative stress in the mammary gland tissue induced by high milk production, in particular in highly productive dairy cows; several studies have focused on the relationship between adult mammary stem cells and the oxidative state of the gland. The oxidative state of the mammary gland appears to be involved in the initial development and metastasis of breast cancer through interference with mammary cancerous stem cells. This review summarizes some links between the mammary stem and oxidative state of the gland.

## 1. Role of Adult Stem Cells in Bovine Mammary Gland Biology

The complex and extensive transformations cyclically shown by the mammary gland are linked to the presence of cells with stemness, or as a better definition, only to stem cells that have a proliferative capacity to drive a significant increase in the cell proliferation rate, which determines cyclic processes of mammary gland remodeling during pregnancy [1]. This particular type of cell probably plays a role in the substitution of epithelial cells that exfoliate in the lumen of the ducts during lactation. Different types of progenitor cells have been characterized, partially addressed toward a mammary phenotype. They are organized according to a well-defined hierarchy: the most primitive cells are those defined as adult stem cells. These cells give rise to the different types of cells present in the functional mammary unit, the alveolus. The mammary precursors are cells already partially differentiated, and therefore have a lower multipotent capacity but with a large proliferative capacity. Because of activity, their total number in mammary tissue is higher. 

In the bovine species, during postnatal life, the mammary gland begins to develop after a first quiescent phase, a process with an initial formation of compact and branched ducts immersed in an environment composed of loose connective tissue. The subsequent elongated growth of these formations occurs under a coordinated regulation that also determines the branching and propagation process of the terminal ductal units and the proliferation of the connective tissue that slowly spreads among the adipocytes forming the mammary fat pad. When the animal reaches sexual maturity, mammary development stops and minor changes take place during the cyclical repetition of the estrous and luteal phases, due to the simultaneous hormonal changes, in particular related to the progesterone and estradiol concentrations. 

However, during pregnancy, the mammary gland, under the influence of the hormonal milieu essentially composed of progesterone, undergoes a powerful development immediately after fertilization and ends with delivery. At the tissue level, the mammary epithelium proliferates enormously through the constitution of secondary branches, and then tertiary ducts, with an expansion of the nonfunctional alveolar structures, end with a definitive maturation of the cellular phenotype [2,3]. This crucial remodeling aims to increase the total amount of functional cells throughout the terminal differentiation. The mature differentiation occurs with the expression of a specific protein, in particular β-casein and α- and β-lactoglobulin, which are the specific protein components in milk. The possible association between the pool of primitive cells and the total mass of functional parenchyma of the mammary gland is of great interest, as the yield of milk is correlated with the development of the gland.

## 2. Recent Insights for Bovine Mammary Stem Cells Characterization

Although most of the data for the hierarchy and the behavior of resident progenitor cells in the mammary gland have been mainly collected in human and murine species, efforts were made to identify and study these cells even in bovines [4,5]. The existence of a population of adult stem cells has been reported and a method based on flow cytometry to isolate different subpopulations of progenitors has been proposed [6]. Another research group described the phenotype of the different populations of mammary progenitors according to the expression of surface antigens [7].

## 3. Stem Cells Regulation Interferes in Milk Production

Given the close correlation between the quantity of milk produced and the number of active secreting cells, the more we know about the mechanism that maintains the cellular functionality of the secreting cells, the more we will understand about increasing milk in time, defined in dairy science as the persistence of lactation. It has been demonstrated in the lactation curve of dairy cows that after an initial increase in milk production induced by enrichment of the secretory capacity of the luminal cells, a constant phase of milk production is observed followed by a slow decline in productivity correlated with a slow and steady reduction in the number of epithelial cells. This is the result of an unbalance ratio between the increasing apoptotic rate and a slightly lower proliferative activity of the mammary epithelium, which is not able to support a constant turnover of cells secreted throughout lactation [8]. In this context, modifications focused on stem cells activation and progenitors proliferation may have a real effect on the cow’s productivity: dairy cattle management aims to fertilize the cow about three months after parturition, this minimizes the time during which the cow is unproductive, since subsequent lactation can be initiated shortly after finishing the previous one. The time between two lactations is termed the dry period. Of course, the shorter this period, the less unproductive the animal is. When the lactation period stops, the mammary gland undergoes an involution process that leads the organ to a quiescent phase before the next pregnancy. However, in the dairy cow production chain, this does not occur since a new pregnancy is concomitant with the lactation condition. The pregnancy determines a hormonal milieu that contrasts greatly with the end of lactation and strongly enhances cell proliferation and mammary morphogenesis [8]. Thus, a condition of regenerative involution occurs that is associated with the dry period, in which the mammary gland does not undergo organ remodeling, but rather massive cell turnover. This event is essential to ensure the significant production of milk in the terminal part of lactation. It is uncertain how this high cell proliferation rate is supported by a niche of undifferentiated mammary cells that, however, are activated by a specific hormonal milieu that assures correct cellular metabolism.

In this scenario, the dry period is a crucial phase for dairy cow management and the regulation of homeostasis in the stem/progenitor cell compartment is fundamental. The important metabolic stress experienced by cows during this great energy expenditure is lactation and the progressive aging of the animals causes a reduction in progenitor activity and, consequently, a lower productivity.

## 4. Stem Cells in Milk

Milk is a source of immune and epithelial cells. Quantitative analyses include the somatic count, which does not interpret the cell type but only the level of cell bodies present. Normally, stem cells or precursors are isolated and characterized from a phenotypic and functional point of view by the dissociation of breast tissue. For the evaluation of the activation and expression patterns of these cell types over time in an animal, this approach is difficult to apply, as biopsy is required. The progenitor cells were reported to be directly isolated from milk in humans [9,10]. The authors showed that in human milk, a cellular population of epithelial origin (with the expression of different types of cytokeratins) is able to express cellular markers characteristic of primitive cells, including nestin and p63. These specific cells are able to generate cell colonies with different terminal markers of breast tissue that expand through accelerated proliferative activities.

In bovine milk, the frequency of putative primitive or mature differentiated epithelial cells has been reported [11] and these cell subpopulations are differentially expressed according to the lactation phase. Since the mammary epithelium is structured as a bilayer, two different cell lineages are characterized by the expression of specific markers. Luminal cells are cytokeratin-enriched in CK18 with low expression in CK14, whereas myoepithelial cells demonstrate the opposite. This alveolar organization is well conserved among different mammalian species, including humans [12], mice [13], goats [14], and bovines [2]. Another important cell surface marker is CD49f, a component of the laminin-1 receptor, which is expressed in the outer layer of the alveolus. A high CD49f expression has been associated with myoepithelial progenitors and/or mammary adult stem cells [12,15]. The characterization of epithelial somatic cells in bovine milk has shown that a specific CK14^+^/CK18^−^ subtype of epithelial cells increases toward the end of lactation. The cause for this increase may be the gradual exhaustion of the inner secreting cell layer at the end of lactation. Thus, this epithelial cell subpopulation may be considered the signal of a reduction in mammary efficiency. This reduction could have a physiological aspect, but different factors should be considered, such as mammary gland pathologies and management related to milking. 

The role of factors that counteract the action of reactive oxygen species (ROS) at the mammary level was underlined by the discovery of various substances with antioxidant activity present in colostrum and in cow’s milk [16,17], which directly play a role in the epithelial alveolus, especially at the beginning of lactation. Distress can affect the somatic cell epithelial subpopulations, maybe indicating that animal welfare affects mammary gland functionality with a lower expression in CD49f^+^ and K18^+^ cell populations [18]. 

A report proposed that ROS regulate cell differentiation in mammary glands. An increase in this epithelial cell subpopulation was directly correlated with the loss of milk-producing capacity of the mammary gland during the last part of lactation [11], leading to the hypothesis that this cell population could be a biomarker of the production efficiency of the mammary gland.

The presence of CD49f^+^-enriched cells, even in low amounts, may be associated with a decrease in the myoepithelial compartment, indicating a changing myoepithelial genetic program [18,19]. CD49f is an integrin that is a component in a feedback circuit that interferes with the myoepithelial phenotype in mammary epithelial cells in different species such as human and mouse [20,21]. This led to the hypothesis that the basal regulatory machinery may be unpaired in myoepithelial cells, and inappropriately engaged in luminal epithelial cells during a loss in tissue functionality. Different distributions of mammary epithelial cell subpopulations, recovered from milk, provide more detailed information on the physiology of mammary glands during lactation and, potentially, may be considered for the evaluation of mammary gland biology. 

A stem cell-like population has been isolated from bovine milk [22], further validating previous studies in human counterparts [10,23,24,25]. This outcome opens new possibilities for the veterinary use of milk as an innovative and noninvasive source of multipotent stem cells, which may have a role in the field of regenerative veterinary medicine [26].

## 5. ROS in Mammary Gland Involution

The mechanism of action that specifically affects mammary gland involution and increases the luminal cells apoptosis rate in the alveoli, with less impact on other compartments such as myoepithelial and ductal cells, is still unknown. The involution process in mammary organoids in culture is stimulated by fresh media withdrawal and is characterized by cellular oxidative stress and expression of activated macrophage marker CD68 [27]. This process can be mimicked by exogenous addition of ROS in cultures without media withdrawal. ROS are chemically reactive molecules containing oxygen produced as a physiological product of oxygen metabolism. At normal levels, ROS participate in the regulation of various physiological events, such as cell proliferation, cell migration, wound healing, and angiogenesis [28]. In mammary gland, cells dissociated from post-involution alveoli were enriched in CD49f, thus mammary precursors or mammary stem cell phenotypes were able to reproduce a complete alveolar structure in subcultures without any significant loss in viability [29]. However, ROS produced by accumulated milk breakdown postweaning were shown to be the cause underlying the selective involution of secretory alveolar luminal cells. The process of involution in a mouse model involves a complex set of fine regulation of molecular and physical events that can be classified into two phases. In the first stage, the accumulation of milk in the alveolar lumen is necessary to initiate the first (reversible) phase during which the secretory cells begin to enter apoptosis. The second phase of involution begins when matrix remodeling enzymes are upregulated. This stage is irreversible. The role of matrix in remodeling development appears to be linked to the function of stromal fibroblasts surrounding the lobuloalveolar network in mice, but not in all species, since humans and bovines do not show the same cellular organization. However, this role is likely to be regulated by epithelial–mesenchymal cross-talk [29,30]. In the animal model, when the involution is still evident, a minor population of lactation-associated cells, which remains at the basal surface, are considered a sort of track for subsequent lactation [27,31]. A negative feedback loop mediated by the mechanical pressure of the filled alveolus regulates milk synthesis and triggers secretory cell apoptosis [32]. 

**Figure 1:**
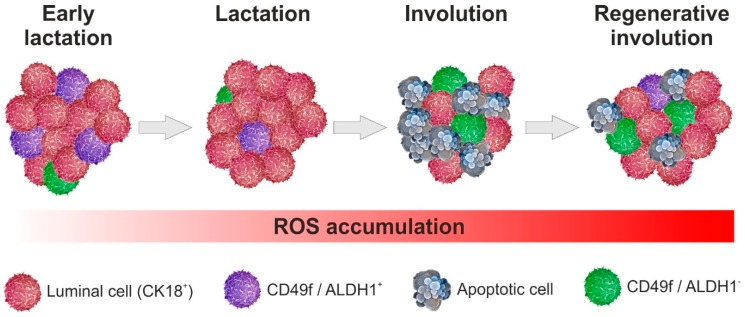
Precursors cell population during regenerative involution in bovine mammary gland.

There are also biochemical mechanisms that coordinate involution in association with the mechanical stimulus. These mechanisms are driven by a component of the milk [33]. Especially for mammary glands, the oxidative stress resulting from excess ROS has been shown to be an initiator of apoptosis [34,35]. A previously report proposed that ROS produced by the breakdown of the accumulated milk are associated with the activation of apoptosis in the first phase of mammary involution [9]. At the onset of irreversible luminal cell death, a particular cell type with a macrophage surface marker, such as CD68, arises in the inner layer cell population. Thus, ROS are a signal to trigger cell death in the ROS-sensitive epithelial secreting cells, whereas the ROS-insensitive population is maintained, and the process ends with the preservation of a signal originating in the CD68 epithelial secreting cell type [9]. According to what has been previously reported, in this phase we find a small population resistant to the apoptotic process that face the luminal cells CK18^+^, that of CD49f^+^/ALDH1^−^ which is considered very close to the characteristics of mammary stemness. Figure 1 shows the different mammary cells affected by ROS during the regenerative involution in bovine mammary gland.

Other pathways are involved, in addition to the ROS effect, in mammary gland involution since it becomes irreversible after a specific timepoint post-initiation, even when ROS are not present. This temporal switch has been previously implicated both in vivo and in vitro in other models [36,37]. The limit that separates ROS-induced reversible activation and the irreversible completion of breast invasion seems to be marked by a positive CD68 population. The outer cell basal population that resists after involution is significantly less sensitive to ROS-associated cell death than the inner luminal cell population. Basal cell populations that still survive after a complete involution have been proposed to be responsible for giving rise to sequential healthy lactations. 

In dairy cattle, there are several reports evaluating the role of oxidative stress or its amelioration by supplementation of polyunsaturated fatty acids (PUFA) substrates or precursors. In these trials, it is difficult to properly measure the oxidative stress status to deeply observe the role of ROS in mammary gland functionality. These issues have been previously discussed [38] and further investigations should provide more precise assessments or macromolecular damage products, in particular for lipids and proteins. For example, PUFAs are highly susceptible to peroxidation during oxidative stress; thus, their specific peroxidation metabolites have been proposed for accurate oxidative stress diagnosis in dairy cattle. In this sense, the isoprostanes are a category of interesting molecules for a finer analysis of the oxidative, which are already considered in oxidative stress in human medicine, in particular for cardiovascular problems and sepsis [39].

## 6. Role of ROS in Breast Cancer Stem Cell Pathology

Although not particularly widespread in bovines [40] but important in dogs, cats, and humans, the presence of tumor pathologies affecting the mammary glands is correlated with the analysis of breast cancer stem cells and ROS appear to exert a role. Since intracellular ROS and the redox balance have been implicated in mammary epithelial cell growth and differentiation [9,41], studies indicate that ROS activity plays a significant role in the process of epithelial–mesenchymal transition, which is crucial for metastatic mammary neoplasia [42,43,44]. ROS are produced in large quantities in response to environmental stressors (experimentally determined by radiation, exposure to heat or ultraviolet (UV) radiation) and these interfere with some macromolecules, such as lipids, proteins, and DNA, also determining cell death [45]. These cancer stem cells have been identified in many neoplastic diseases and have been linked to the development of relapses or metastatic involution of the disease [46]. These cells belong to a niche of stem cells with specific properties such as self-renewal and the development of different tumor cell lines [47]. These characteristics enable this cellular subtype population to determine the serious involution of the neoplasm toward a metastatic or recurrent form [48]. These cancer stem cells are capable of activating the epithelial–mesenchymal transition, a specific metastatic involution [49]. These cells have been discovered to possess a specific regulation of protection against the harmful effects of ROS, such as their elimination and the ability to reduce their production. Finally, in this cellular population, the genes encoding superoxide dismutase, catalase, and glutathione peroxidase, all enzymes particularly involved in the scavenging of ROS are overexpressed [50]. It has been reported that in normal breast stem cells and in a subset of cancer stem cells, at least in mice and humans, lower levels of ROS were measured compared to their cell descendants. These data led us to hypothesize that in different tissues, there are multipotent adult stem cells that conserve that cellular population from any damage induced by the oxidative state either due to endogenous or external causes.

An increase in free radical scavengers would lead some cancer-like stem cells to keep ROS levels low, even if extreme variability is observed in this phenomenon for both adult physiological stem cells and in different cancer stem cell subtypes with possible interference from environmental factors. This subtype of stem cells with low ROS levels could be the result of a quiescent cell population.

## 7. Conclusions

The accumulated evidence of the roles of ROS-driven normal and pathological involution in mammary glands provides insights into opportunities for modulating oxidative status. There is a possibility that through their control, mammary functionality can be modulated. The period of late lactation and the dry phase are of special interest in the field of animal production, and is of primary importance in the dairy cow production chain. Furthermore, another possibility is targeting cancer cells’ ability to detoxify ROS, which have been frequently observed to be upregulated in breast cancer stem cells [51]. 

In conclusion, the role of ROS appears to have been ascertained as potential biomarkers of patophysiological suffering of the mammary gland, as well as targeting the mammary stem cells present in the normal tissue or derived from tumor initiation.
